# Editorial: Women in heart valve disease

**DOI:** 10.3389/fcvm.2023.1150169

**Published:** 2023-03-13

**Authors:** Verena Veulemans, Marie Billaud, Maria Carmo P. Nunes, Claudia Goettsch, Elena Aikawa

**Affiliations:** ^1^ Department of Cardiology, Pulmonology, and Vascular Diseases, University Hospital Düsseldorf, Düsseldorf, Germany; ^2^ Department of Surgery, Division of Thoracic and Cardiac Surgery, Brigham and Women's Hospital and Harvard Medical School, Boston, MA, United States; ^3^ Hospital das Clinicas, School of Medicine, Federal University of Minas Gerais, Belo Horizonte, Brazil; ^4^ Department of Internal Medicine I, Cardiology, Medical Faculty, RWTH Aachen University, Aachen, Germany; ^5^ Cardiovascular Medicine, Brigham and Women's Hospital and Harvard Medical School, Boston, MA, United States

**Keywords:** heart valve disease (HVD), basic research, clinical research, mitral valve, CAVD (calcific aortic valve disease), LV remodeling

**Editorial on the Research Topic** Women in heart valve disease

The present editorial summarizes articles published by women investigators in Frontiers Cardiovascular Medicine, Heart Valve Disease Section, and promotes the work of female scientists with a strong focus on heart valve disease. Even if women have made progress in education and science, they are largely under-represented and constitute only thirty percent of researchers worldwide, a condition known as STEM (Science, Technology, Engineering, and Math) -gap. Long-standing gender stereotypes that are frequently experienced by women may lead to a natural limitation for success, further discouraging high-potential females from entering these areas of research. Even if the presence and visibility of women are successively increasing in the medical area, their contribution to scientific fields usually lags behind men and develops slower. This exclusive collection of articles offers a view on outstanding research performed by female scientists in the field of heart valve disease.

However, ideally, gender should not have any impact on science, and we have to be careful that the empowerment of female scientific work does not end in discrimination against men. Looking back on the previous and current status quo in cardiovascular medicine and science, women still remain under-represented, including leading author positions, scientific or clinical meeting responsibilities, speaking engagements, and principal investigator roles in randomized clinical trials (1–4). Furthermore, women usually receive less research funding and more critical reviews than men (5), favoring a double-blind review process as an optimal and gender-neutral publication strategy in the future. As long as this is not routinely performed, other strategies to break the wheel of gender disparity in cardiovascular science are necessary, such as supporting educational and network platforms, which are also well-accepted for male networks and collaboration. In this context, van Spall et al. (3) provided a helpful roadmap concerning strategies to resolve long-lasting gender disparity, including monitoring key metrics by investigators, institutions, professional societies, industry and funding agencies, and scientific journals.

Regarding this collection of articles and the submission eligibility, the five handling female editors invited female researchers from their network and female scientists through research call/promotion actions on social media (Twitter, LinkedIn). Notably, male researchers were also contacted to provide names for eligible women for a research contribution. All nine articles had females as first authors, while 7 out of 9 articles had a female in senior position. According to availability, the reviewer process was gender-independent and performed mainly by at least one male and one female reviewer. The handling editors made the final decision after the independent review process was finalized. Figure 1 illustrates the field research contribution and gender-related responsibilities within this special issue also involving men during the whole publication process (overall distribution of women/men: 70/30%) but with a clear focus on women in primary positions. However, as female scientists still represent a minority of first and last authors in cardiovascular research, journals should ideally blind and monitor their peer review processes to eliminate gender bias in one or another direction in the future.

**Figure 1:**
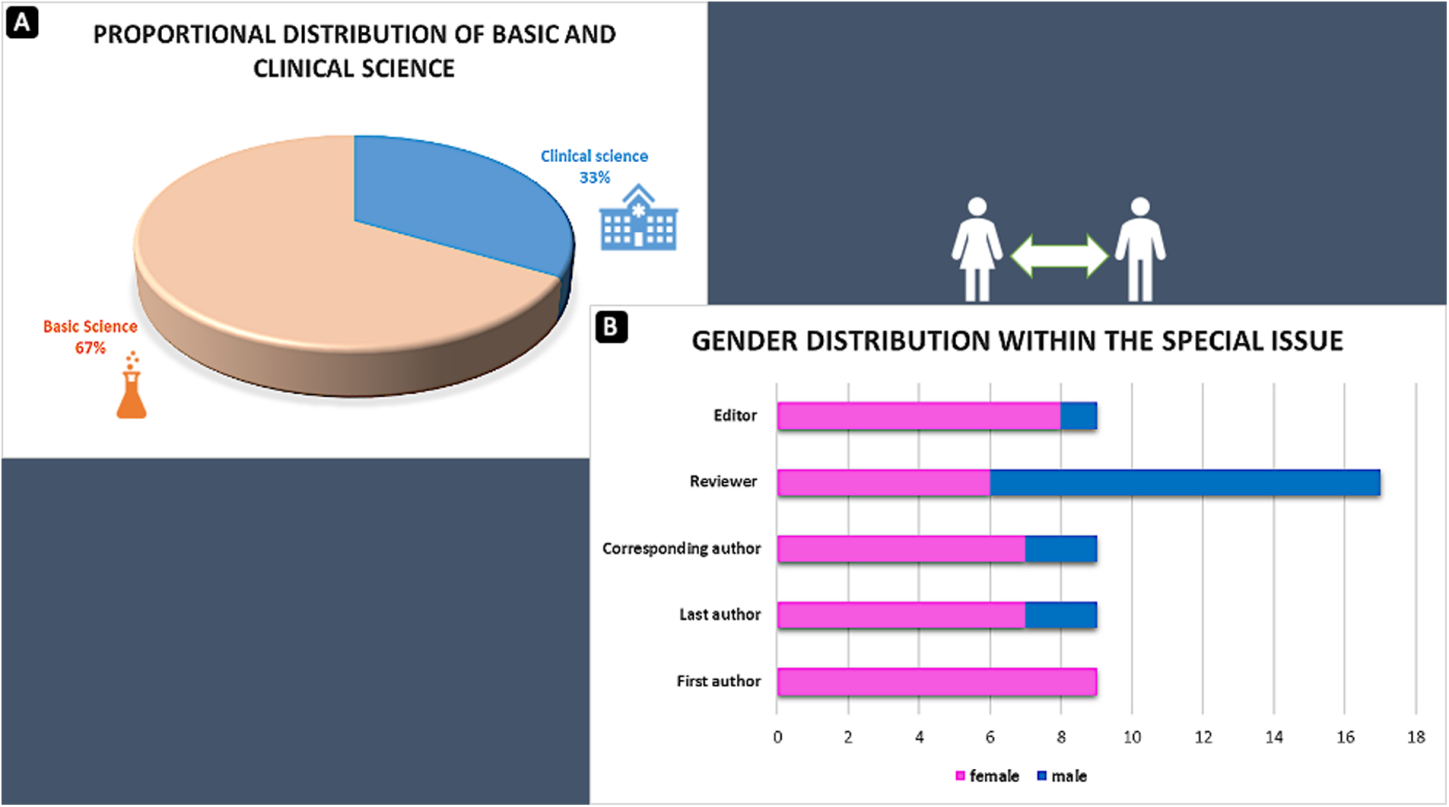
(**A**) Proportional distribution of basic and clinical science. (**B**) Gender-related responsibilities within this special issue.

## Take-home message

The reported research in this section features different scientific considerations within heart valve disease, including basic and clinical research predominantly performed by women, providing an outstanding contribution to future research and facilitating greater diversity in cardiovascular research leadership.
